# A Community-Based, Multidisciplinary Training Program to Improve HIV Services for US-Based African Immigrants: Lessons Learned over 5 Years of Implementation

**DOI:** 10.3390/ijerph23030332

**Published:** 2026-03-06

**Authors:** Chioma Nnaji, Rena C. Patel, Agatha O. Adigwe, Philip G. Day, Maria Fernanda Escobar

**Affiliations:** 1 Multicultural AIDS Coalition, Boston, MA 02119, USA; aadigwe@mac-boston.org; 2 Division of Infectious Diseases, Department of Medicine, Heersink School of Medicine, University of Alabama at Birmingham, Birmingham, AL 35294, USA; renapatel@uabmc.edu; 3 Department of Family Medicine and Community Health, University of Massachusetts Chan Medical School, Worcester, MA 01655, USA; philip.day@umassmed.edu; 4 Institute on Assets and Social Policy, Heller School for Social Policy and Management, Brandeis University, Waltham, MA 02453, USA; mfes7@brandeis.edu

**Keywords:** African immigrants, HIV, provider training, community–academic partnerships, continuing education

## Abstract

African immigrants in the U.S. face disproportionately high rates of HIV, yet existing services often lack alignment with their cultural and linguistic needs. Healthcare providers are rarely trained to address these gaps, contributing to persistent disparities in HIV prevention and care. Traditional in-house trainings for providers often have limited applicability. Collaborative, community-engaged approaches offer an opportunity to build culturally responsive, coordinated care. African Immigrant (AI) Health was a 5-year initiative integrating the Extension for Community Healthcare Outcomes model, Community of Practice model, and Commitment to Change (CtC) evaluation framework. We used an iterative process to improve the AI Health program based on participant feedback. Data were collected through pre/post-CtC questionnaires, case presentations, and discussion groups. CtCs were analyzed thematically and organized using the socioecological model. Over five years, 100+ providers enrolled, with 58 completing the program. Participants represented diverse healthcare roles, with a majority identifying as Black/African American (62%), female (81%), and residing in the Northeast region (57%). A total of 390 CtCs were developed, with the most common themes at the individual level. Most (54.5%) participants partially implemented their CtCs. Participants who implemented their CtCs reported improvements in culturally responsive care. Iterative adaptations enhanced engagement and retention. AI Health effectively supported providers in enhancing HIV care for African immigrants through culturally responsive training. Findings highlight the need for sustainable, longitudinal training models that integrate community expertise and call for policy and structural reforms to address systemic barriers to equitable HIV care.

## 1. Introduction

In 2000, the U.S. Department of Health and Human Services’ Office of Minority Health issued the National Standards for Culturally and Linguistically Appropriate Services (CLAS) in Health and Health Care to guide United States’ (U.S.) healthcare providers and systems in delivering culturally and linguistically appropriate services [1]. The CLAS standards aim to ensure that all patients receive equitable and effective care, regardless of their cultural background or English language proficiency. Adhering to these standards has been shown to improve patient satisfaction, enhance patient–provider communication, and, in the long term, reduce health disparities [2,3,4].

Despite progress under CLAS, critical gaps persist, particularly in addressing complex health issues like HIV. While Black communities account for a significant proportion of new HIV diagnoses in the U.S., African immigrants—a distinct ethnic subgroup—face even greater disparities. Their rates of HIV diagnosis are three to six times higher than the U.S. average, often due to delayed testing and late stage diagnosis [5,6,7]. Additionally, while Black individuals overall represent only 14% of PrEP users, African immigrants experience some of the lowest uptake rates of PrEP [8,9,10].

Healthcare providers working with African immigrants face unique challenges in delivering effective HIV services. Many lack the training and resources to address this population’s specific cultural and linguistic needs, resulting in care that is often inaccessible or culturally misaligned [11]. These barriers are compounded by systemic issues, including limited access to health insurance, fear of immigration enforcement, and a lack of tailored HIV interventions [12,13]. African immigrants face intersecting forms of oppression, such as structural racism, sexism, and xenophobia that exacerbate health disparities [14,15]. Additionally, the cost of care and the complexity of navigating the U.S. healthcare system further marginalize African immigrants, underscoring the need for significant structural changes in U.S. systems, policies, and service delivery models.

There is a significant lack of research and interventions defining best practices for HIV services tailored to this population. This gap is primarily due to insufficient attention to the differences among Black ethnic subgroups in the U.S., enumeration by country of birth in data collection [16], and a lack of dedicated funding to explore and address these disparities effectively. Integrating community partners with lived experience into the design and delivery of HIV services can enhance care by addressing stigma, improving cultural and linguistic appropriateness, and fostering trust within African immigrant communities. Much of the existing HIV literature for African immigrants emphasizes the importance of community-defined strategies, including the use of culturally and linguistically tailored interventions developed in collaboration with the population [17]. Clinical-community partnerships have also proven successful by offering HIV testing in non-clinical settings, such as churches and community venues, and incorporating cultural practices to reduce HIV-related stigma [12]. Furthermore, community engaged research has been steadily growing within the field of African immigrant health, with a primary focus on documenting the epidemic and identifying HIV-related needs and challenges [18,19].

Such community-centric collaborations are also needed for provider training and continuing education focused on improving the health outcomes of African immigrants. To effectively close the gap in HIV service delivery, healthcare systems must prioritize collaborative training efforts with established and trusted community-based organizations. Traditional in-house trainings for providers often have limited applicability because they lack the context of lived experiences and have limited consideration of social determinants of health. We propose that collaborative approaches engaging both providers and community organizations in bi-directional learning can eventually provide a foundation for sustainable, culturally appropriate HIV care that is well-coordinated across community–clinical settings and meets the needs of African immigrant populations.

This paper outlines the development of a community-led provider training and continuing education program designed to improve HIV care for African immigrants. We describe the program’s iterative design and implementation process over 5 years. It provides lessons learned to guide others in creating similar programs.

The University of Massachusetts Chan Medical School IRB determined that this project did not constitute human subjects research under DHHS and FDA regulations (IRB ID: STUDY00002895); therefore, IRB review and approval were not required.

## 2. Materials and Methods

### 2.1. Development of AI Health

Our program, African Immigrant (AI) Health, was initially designed by C.N. (first author on this article) and further developed with Dr. Bisola O. Ojikutu, MPH, FIDSA (B.O.O), who served as a co-director in its early stages. Both are second generation Nigerian American and have years of experience providing HIV services to African immigrants in Massachusetts. C.N. started in the HIV field as a Community Health Worker and founded in 2000 the Africans for Improved Access (AFIA) program at the Multicultural AIDS Coalition. B.O.O. is a board-certified physician in internal medicine and infectious diseases and practices HIV medicine within the Infectious Disease Division at Massachusetts General Hospital.

AI Health was built on the community–clinical relationship that C.N. and B.O.O. established while working together to serve African immigrants living with HIV, particularly individuals who were not engaged in care. This relationship emphasized the importance of bridging gaps in the continuum of care by addressing cultural, linguistic, and systemic barriers. The co-directors’ lived realities as second-generation Nigerian immigrants and their frontline experiences shaped the initial program design and priorities. The foundation for AI Health also drew on the long-standing work of MAC/AFIA in documenting and responding to HIV disparities among African immigrants [20]. In 2021, the program’s faculty changed, and R.C.P. (co-author on this article) replaced B.O.O. as the co-director. R.C.P is an infectious diseases/HIV physician and researcher and a second generation South Asian American, which provides a relevant immigrant lens to her work with African immigrant communities in the U.S. Over the 5-year grant cycle, iterative adaptations were also made based on participant feedback across cohorts, which included providers and people living with HIV who also identified as African immigrants.

### 2.2. AI Health Program Goal and Structure

The primary goal of AI Health was to enhance HIV services by improving community–clinical linkages and systems of care for African immigrants living with or vulnerable to HIV. Objectives were for participants to: (1) learn promising practices and strategies to provide HIV services to African immigrants; (2) discuss prevention and treatment advances for HIV and related diseases; (3) share challenging cases and gain insight from colleagues; and (4) connect with HIV providers across the U.S. to strengthen HIV services engaging African immigrants.

AI Health integrates two learning models, Extension for Community Healthcare Outcomes (ECHO) and Community of Practice (CoP), and an evaluation framework, Commitment to Change (CtC). The ECHO model is an evidence-based approach designed to democratize knowledge and amplify best practices through virtual communities. The model uses a hub-and-spoke knowledge-sharing network, where experts at the “hub” provide mentoring and guidance to providers in community settings, the “spokes” [21]. This approach allows local providers to manage complex conditions within their communities, improving patient and community outcomes by increasing local capacity [22]. The ECHO model has been adapted to address a wide range of health conditions, including cancer screening, palliative care, mental health, chronic pain, and opioid use disorder [23,24,25,26,27,28]. Case presentations are an essential aspect of the ECHO program model to help participants navigate complex client cases through discussing real-life scenarios and exchanging evidence- or practice-based methods.

A CoP is a group of individuals who share a common interest or profession and engage in collective learning and knowledge sharing over time [29]. The CoP model emphasizes the importance of learning in a social context, where members participate in collaborative activities, share resources, and engage in discussions to build and expand their professional skills [30]. By fostering ongoing interaction and shared experiences, CoPs help members stay informed about new developments in their field and innovative practices.

CtCs are used in medical education as a reflective practice approach that supports the application of learning in real-world clinical settings [31]. In addition to individual-level benefits, CtCs can influence organizational change by encouraging healthcare professionals to implement evidence-based practices, improve team communication, and address systemic barriers in their workplaces [32,33].

The program underwent iterative development over a 5-year funding cycle. Leveraging participant feedback, the program team refined its structure and content to better meet the needs of participants and the changing dynamics within the HIV field and broader society. Table 1 shows how the program changed over the 5-year period with specifics about structure, content, and evaluation metrics.

The most recent version of the AI Health program implemented from 2022 to 2024 consisted of a virtual 2-day, 6 h per day intensive followed by eight weekly 1.5 h virtual ECHO sessions. Presenters, panelists, and facilitators for each session were largely from African immigrant backgrounds. Day one familiarized cohort participants with the program’s format, established group agreements, and included sessions on two key topics: the epidemiology of HIV among African immigrants and HIV care and treatment. Day two covered the remaining topics through interactive presentations or panels, focusing on health equity and structural oppression; African LGBTQ health; immigration; the impact of hepatitis B; stigma and cultural myths, barriers, and facilitators to care; mental health; and women’s health and reproductive care. Both days included breaks and lunch time. At the end of each day, participants completed a pre-CtC survey asking them to brainstorm CtCs based on the information presented. The first weekly ECHO session was dedicated to finalizing 1–2 CtCs per participant. Each subsequent ECHO session included an opening activity, group check-ins on CtC implementation, and a structured process for 1–2 case presentations using the ECHO framework.

We recruited AI Health participants using a flyer distributed through staff networks, email campaigns, social media, previous participants, and the New England AIDS Education and Training Center (NEAETC) listserv. Individuals completed a SurveyMonkey (Momentive Inc., San Mateo, CA, USA) application to gather demographic information, explain their interest in the program, detail their organizational affiliations and roles, experiences serving African immigrants, and identify specific topics they were interested to explore. Eligible participants were selected by program staff based on criteria that included working in HIV, diversity in profession, geography, and prior experience working with African immigrants. Selected participants were required to register in NEAETC’s Learner Education and Practice Portal (LEAPP)©, an online registration system, before the first session (University of Pittsburgh, Innovation Institute, Pittsburgh, PA, USA).

### 2.3. AI Health Program Tools

We used several data collection tools to track the progress and engagement of participants, as well as to assess the effectiveness of the program. See Table 2. These tools included templates adapted from Project ECHO, the coordinating center for ECHO-based programs at the University of New Mexico Health Sciences Center, as well as custom-designed tools created by NEAETC and AI Health staff.

The pre- and post-program Commitment to Change (CtC) questionnaires were developed specifically for AI Health, adapting questions from Wakefield et al. (2003) [31] and Holmboe et al. (2010) [34] and incorporating required items from the funder, New England AIDS Education and Training Center (NEAETC), and the continuing education provider.

### 2.4. Continuous Program Improvement Process

Over the 5-year period, different evaluation consultants were engaged. Each consultant employed a mixed-methods approach to examine participants’ perceptions of the program and recommendations for ongoing improvements [35,36].

Quantitative data were collected through a participation tracker and pre- and post-CtC questionnaires. Analyses were performed in STATA (Release 17; StataCorp LLC, College Station, TX, USA) [37], while Excel was used to summarize the results into tables. These analyses included measures of central tendency (mean), measures of variability (standard deviation), and frequency distributions (counts and percentages). Qualitative data were gathered through virtual discussion groups conducted at the end of each cohort. The number of discussion groups varied (depending on number of program participants) from one to three across cohorts. Participants in these sessions received a $50 or $100 gift card (depending on the program budget) as a token of appreciation. The audio recordings were transcribed verbatim or by Otter.ai (Otter.ai, Inc., Mountain View, CA, USA) [38], then coded and thematically analyzed using ATLAS.ti (ATLAS.ti Scientific Software Development GmbH, Berlin, Germany) [39] or NVIVO (QSR International Pty Ltd., Doncaster, VIC, Australia) [40]. An evaluation consultant conducted the coding and developed the resulting themes.

For this paper, we employed a mixed methods approach to triangulate findings from the various data collection tools. Quantitative data were summarized over the 5-year period using STATA [37] while Excel spreadsheets were used for data organization. For qualitative data, we conducted thematic analysis to group individual CtCs into larger, overarching themes [41]. Post hoc, we applied the socioecological model as an organizing framework [42]. This analytical approach allowed us to synthesize findings and gain deeper insights into the program’s effectiveness and impact for quality improvement and program development purposes.

## 3. Results

### 3.1. Participant Demographics

Across the five years, more than 100 individuals enrolled in AI Health (Table 3); however, a total of fifty-eight individuals completed the program in its entirety. The majority of the program participants self-identified as Black/African American (*n* = 36, 62%). Most were women (*n* = 47, 81%) and resided in the Northeast region (*n* = 33, 57%). Participants represented a broad range of professions. More non-clinical providers participated, such as social workers and case managers, compared to clinical providers, such as physicians and nurses.

**Table 2:** AI Health Data Collection Tools.

Data Collection Tools	Description
LEAPP© Registration Form	An online system managed by NEAETC to collect participant information such as demographics, professional background, types of HIV services provided to HIV clients (direct and indirect), and level of Ryan White HIV/AIDS funding. LEAPP© is a product of the University of Pittsburgh, offered as a licensed software-as-a-service (SaaS) through the University of Pittsburgh Innovation Institute, developed by Professor Linda Rose Frank, PhD, MSN, ACRN, FAAN, and Matthew Garofalo, MBA, MS-MIS, from the Department of Infectious Diseases and Microbiology, School of Public Health, University of Pittsburgh.
Participant Tracker	An Excel document that captured participants’ job-related information, such as job title and credentials, tracked attendance, and monitored the completion of data collection forms. It was updated weekly after each session.
Client Level Case Presentation Form	The form is completed by participants to present details about client cases during ECHO sessions. It includes comprehensive sections to capture patient demographics, migration and legal status, HIV-related information (e.g., diagnosis, treatment adherence, viral load), mental health and social factors (e.g., housing, employment, substance use), cultural beliefs, and community supports. All information was de-identified to maintain confidentiality.
Organizational Level Case Presentation Form (developed in 2024 because of participant feedback)	The form documented challenges faced by organizations in providing HIV/AIDS services to African immigrant communities. It collects detailed information about the organization, including its mission, services, staff composition, and relationship with the African immigrant community. The form also explores community demographics, cultural awareness, and strengths, while identifying specific challenges or conflicts affecting service delivery. All information was de-identified to maintain confidentiality.
Pre-Commitment to Change (CtC) Questionnaires	Pre-CtC survey captured participant commitments. Participants identified 1–3 concrete, measurable changes and reported on their level of motivation and anticipated difficulty in implementing each CtC. Administered using SurveyMonkey.
Post-Commitment to Change (CtC) Questionnaires	Post-CtC survey captured participants’ overall progress with their CtCs. Participants reported on implementation status and reasons for partial or no implementation. Additional questions were asked about satisfaction with the overall AI HEALTH program. Administered using SurveyMonkey.

**Table 3:** Demographics of Participants, Cohort 1–Cohort 4.

		Cohort (Year)				
Characteristics	All	Cohort 1 (2020)	Cohort 2 (2021)	Cohort 3 (2022)	Cohort 4 (2023)	Cohort 5 (2024)
	N	n	n	n	n	n
Participant Enrollment	115	20	17	30	29	19
Completed Program	58	13	8	17	9	11
Didn’t Complete Program	57	7	9	13	20	8
**Participants Who Completed Program**	N (%) ^a^	n (%)	n (%)	n (%)	n (%)	n (%)
**Race/Ethnicity**						
White	14 (24)	5 (38)	4 (50)	2 (12)	2 (22)	1 (9)
Black or African American	36 (62)	8 (62)	2 (25)	12 (71)	7 (78)	7 (64)
Asian	1 (2)	0	0	0	0	1 (9)
American Indian/Alaska Native	0	0	0	0	0	0
Native Hawaiian or Other Pacific Islander	0	0	0	0	0	0
Other	0	0	0	0	0	0
Choose not to disclose	1 (2)	0	0	0	0	1 (9)
Multiple Races	2 (4)	0	1 (13)	0	0	1 (9)
Missing	4 (7)	0	1 (13)	3 (18)	0	0
Latino/a	5 (9)	1 (8)	2 (25)	1 (6)	0	1 (9)
**Gender**						
Woman	47 (81)	11 (85)	7 (88)	11 (65)	8 (89)	10 (91)
Man	8 (14)	2 (15)	0	4 (24)	1 (11)	1 (9)
Missing	3 (5)	0	1 (13)	2 (12)	0	0
**Region**						
Northeast	33 (57)	7 (54)	5 (63)	11 (65)	3 (33)	7 (64)
Midwest	3 (5)	0	0	1 (6)	1 (11)	1 (9)
South	17 (29)	6 (46)	0	3 (18)	5 (56)	3 (27)
West	5 (9)	0	3 (38)	2 (12)	0	0
**Profession**						
Clinical Provider	13 (22)	5 (38)	2 (25)	1 (6)	2 (22)	3 (27)
Social Worker, Case Manager and Community Worker	28 (48)	5 (38)	4 (50)	10 (59)	4 (44)	5 (45)
Clinical Admin or Leader ^b^	1 (2)	0	0	1 (6)	0	0
Non-Clinical Admin ^c^	2 (3)	1 (8)	0	1 (6)	0	0
Other Public Health Professional	14 (24)	2 (15)	2 (25)	4 (24)	3 (33)	3 (27)
**Direct Interaction with Clients**						
Yes	37 (64)	9 (69)	6 (75)	10 (59)	5 (56)	7 (64)
No	13 (22)	4 (31)	0	5 (29)	1 (11)	3 (27)
Missing	8 (14)	0	2 (25)	2 (12)	3 (33)	1 (9)
**Provide Services Directly to Clients with HIV ^d^**						
Yes	30 (52)	9 (69)	5 (63)	8 (47)	4 (44)	4 (36)
No	7 (12)	0	1 (13)	2 (12)	1 (11)	3 (27)
Missing	21 (36)	4 (31)	2 (25)	7 (41)	4 (44)	4 (36)

^a^ Percentage not always equal exactly to 100 due to rounding. ^b^ Clinical administrator represents practice administrator or leader, such as a chief executive officer or nurse administrator. ^c^ Non-clinical administrator includes clergy or faith-based professional. ^d^ Results for this category should be interpreted with caution due to the high proportion of missing values.

Across the 5-year period, non-completion was mostly due to participants not having the time to fully engage in the program. For the last 3 years, participant drop-out mostly occurred after the two-day intensive. The level of participation over the years fluctuated with an average of 8 participants per cohort. The highest number of participants who completed the program was in 2022, cohort 3 (*n* = 17).

Participants decided to participate in the AI Health program for a variety of reasons, such as wanting to gain awareness and understanding about the lived experiences of African immigrants and the opportunity to learn from others. One participant (cohort 2, 2021) expressed that she “felt possibly stuck in [her] own ethnocentric view.” She wanted to expand that view to understand how immigrants see health care, given their very different experiences. There were also a few participants who joined the program because it was recommended by previous participants.

### 3.2. Program Evaluation and Iterative Program Changes over Time

Overall, participants described the AI Health program as an enjoyable experience and excellent learning opportunity that positively impacted their services to African immigrant clients, as well as other immigrant clients. Most found the case presentations, activities supporting participants to connect with each other (e.g., music, icebreakers, contact list), and shared resources (e.g., session recordings, articles) valuable. One participant (cohort 4, 2023) stated, “I have already recommended this training to my colleagues! This training offered so much information and real examples of the barriers other clinicians face with this population. The case studies were the most beneficial part of the training for me because the group was able to come together and problem-solve. I really enjoyed the community and collaboration of the group.” Another participant (cohort 1, 2020) shared, “knowing that you are not alone in your challenges and knowing that you were not failing as a professional because you could not solve all your clients’ “problems” was valuable.”

Participants emphasized the need for more support in engaging and building relationships with African immigrant communities. In addition, many participants cited limited time as the primary challenge for completing activities and discussions, both during the two-day intensive sessions and the weekly follow-up sessions. Due to participant feedback, changes were made to the structure and content over the years.

Program Structure: Initially, we started AI Health as 12 biweekly sessions over 24 weeks. Some participants, mainly clinical staff, were challenged with committing to the program due to demanding work schedules. Hence, after several iterations, we concluded on a 2-day, 8 h intensive followed by eight weekly 1.5 h ECHO sessions to discuss CtC progress and case presentations. Although we still experienced attrition after the initial two-day intensive, participants were generally more willing to engage with the follow-up sessions than in previous iterations.

Commitment to Change (CtC): During initial iterations of the program, participants often committed to three or more CtCs, which made it challenging to achieve even partial implementation. To address this, we introduced several changes to ensure CtCs were more concrete and manageable, resulting in higher levels of implementation over time. As part of the program orientation during the two-day intensive, we provided examples of CtCs to clarify their intent and offered clearer instructions. Additionally, we limited participants to selecting up to three CtCs and encouraged them to focus on actionable items, which sometimes required breaking them into smaller, manageable steps.

Topics for Presentations: Participants suggested that the program include topics that are increasingly relevant to the current healthcare landscape and pressing for them in their day-to-day work with African immigrants. One participant (cohort 3, 2022) said, “The only one that I felt we could get maybe more time on was people who are in the LGBT community as well as in the African immigrant community.” Overtime, several of the topics were added to the two-day intensive, including LGBTQ health, immigration, COVID-19, and telehealth.

Structure of ECHO Sessions: Time was limited during the ECHO sessions to delve into case presentations. To address this, the sessions were restructured to have case presentations at the beginning. This adjustment helped sustain participant engagement and allowed more time to discuss multiple case presentations per session.

Case Presentation Forms: Participants who were not clinicians or who did not work in a clinical setting were challenged in completing case presentations. It was seen as “too formal.” One participant (cohort 2, 2021) did not even look at the form because of this reason. Hence, program staff (led by clinical co-directors) offered individualized support to guide participants through the form completion process prior to sessions and eventually developed an Organizational Level Presentation Form.

While the revised structure improved feasibility and retention, we lost some of the time that previously allowed for organic relationship-building and between-session reflection evident during the longer formats. We mitigated this by (1) allocating dedicated time for CtC check-ins at each follow up ECHO session, (2) narrowing CtCs to smaller, feasible steps, and (3) providing recordings, slides, and resource sheets for asynchronous reinforcement.

### 3.3. Commitments to Change (CtCs)

A unique element of the AI Health program was the CtC process, in which participants identified concrete changes they planned to implement in their practice. Overall participants believed the CtCs were a helpful tool. One participant (cohort 3, 2022) said, “My experience was really wonderful, identifying what I could change, identifying how to change it, and then implementing that change. And then, now continuing it. It’s been a couple of months since we’ve ended and it has been really helpful.” For some participants, it was challenging to choose a CtC that could be implemented in the span of several weeks. Participants in non-client-facing roles found it challenging to create CtCs relevant to their work. Additionally, power dynamics within workplaces hindered some participants from developing or implementing CtCs requiring structural changes.

From 2020 to 2024, a total of 390 CtCs were developed by participants. We identified 20 distinct themes across the socioecological model in our thematic analysis of participants’ CtCs. Table 4 shows the socioecological model levels and specific CtC themes as illustrative examples.

Table 5 reports on the frequencies of all CtCs identified by participants across five socioecological model levels: individual (*n* = 172), interpersonal (*n* = 80), institutional (*n* = 65), community (*n* = 38), and public policy (*n* = 8). The most frequently selected themes were at the individual socioecological level, with knowledge building accounting for 13% (50 CtCs) and attitude change comprising 11% (43 CtCs). Public policy-level themes were the least represented, with policy change and advocacy each accounting for only 1% (4 CtCs).

**Figure 1:**
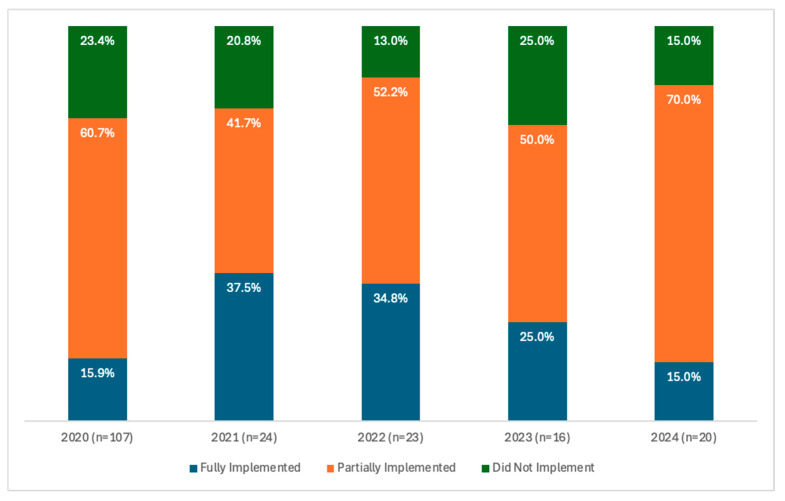
CtCs Implementation for the Period 2020–2024.

Figure 1 only includes implementation data for the CtCs each participant focused on for the duration of the program across cohorts. The 2021 cohort had the highest level of CtC implementation at 37.5%. The average number of participants who fully implemented their CtCs was 25.34%. Those who partially implemented their CtCs were highest in the 2024 cohort at 70.0% with an average of 54.48% across all cohorts. Those who did not implement were highest in the 2023 cohort 25.0% with an average of 19.34% across all five years.

Participants identified several barriers to implementing CtCs, including lack of time (ranging from 38.5% to 50% across cohorts), systems or logistical barriers in their practice, and the need for improved knowledge or skills. Although some participants faced barriers to fully implementing their CtCs, those who were able to do so reported significant positive impacts on their practice.

## 4. Discussion

Over a 5-year period, we engaged in an iterative process to design and implement the AI Health program. AI Health was successful in supporting clinical and social providers—and increasingly program managers and administrators—from across the U.S. in improving HIV services for African immigrants. This program addressed a significant gap in existing training initiatives by focusing specifically on the unique HIV-related needs of African immigrants, accounting for differences in ethnicity, language, culture, and immigration status which are factors often overlooked when serving Black/African American communities. In addition, it emphasized a community–clinical linkage approach to improve continuity of care and ensure that services address the core sociocultural needs of African immigrants. Nonetheless, we faced challenges, ranging from participant retention to determining an effective program format. These experiences offer valuable lessons for other program leaders to apply in their education and training initiatives.

The AI Health program integrated several well-established education and training models. The CoP model created a safe and collaborative environment where participants could openly share challenges, learn from one another, and collaborate beyond the AI Health sessions to support clients. We think a community-based organization, instead of a healthcare or academic institution, leading the program particularly engendered this sense of camaraderie. Our approach reinforced a collective responsibility to address the whole spectrum of barriers at all socioecological levels to providing effective HIV care for African immigrants.

The ECHO model facilitated real-time case-based learning and nurtured leadership among participants. Participants shared that the strategies discussed as part of the case presentations were helpful in motivating them to think differently about the issue and address it with more cultural humility.

The CtC process was important for encouraging participants to implement the changes discussed during sessions, ensuring that the knowledge gained translated into actionable improvements in clinical and organizational practices. Also, the iterative and participatory design allowed for continuous adaptation of both structure and content. This deliberate approach supported meeting the diverse and changing needs of participants who make up the HIV workforce. Admittedly, CtCs that go beyond the interpersonal level, to institutional- and policy-level changes are even harder to address in a program such as AI Health. However, as the field of public health increasingly recognizes structural barriers—such as systemic racism—that hinder optimal health outcomes, there is a pressing need to amplify efforts that actualize change at structural and policy levels.

There was consistent interest in the program. We received 40 to 50 applications each year. Participation was even sustained despite the COVID-19 pandemic occurring during the 5-year funding cycle. Ultimately, 58 diverse participants, including social workers, community health workers, nurse professionals, physicians, and public health administrators were served by the AI Health program. There was a shift in participants’ professional backgrounds over time, particularly in 2022, 2023, and 2024. The program attracted an increasing number of administrators and program managers seeking support in developing more inclusive HIV education, outreach programming, and organizational policies. To encourage retention, Continuing Education (CE) credits were incorporated from the outset, consistent with strategies shown to be effective in previous NEAETC-funded programs. However, attrition still occurred primarily due to limited time for participants to fully engage past the 2-day intensive sessions. Future iterations of AI Health could further reduce attrition by integrating additional strategies such as employer-supported protected time. This serves as a benefit to employers because it supports staff in learning and retaining skills that can strengthen service delivery and improve organizational capacity. Other strategies include AI Health staff offering multiple scheduling options and modular asynchronous content.

An unexpected outcome was that participants were able to connect during sessions to serve clients across states. For example, one participant was able to connect their client to a virtual support group facilitated by a provider in another state (due to stigma, the client did not want to attend a local support group). Another example is that a participant was able to support their client in moving to another state and ensuring access to medication and care because of another participant in the cohort. In addition, professional partnerships arose. For example, two peers connected to apply for funding to establish a national network for African immigrant women living with HIV [43].

In achieving these successes, we also encountered significant challenges. One challenge was the complex Continuing Education (CE) process. As a grassroots community-based organization with a long-standing HIV program engaging African immigrants [20], the Multicultural AIDS Coalition was ideally positioned to implement a provider training program. However, our lack of formal affiliation with an academic institution limited our access to CE certification. The costs associated with obtaining certification annually and changing level of requirements were significant obstacles. Ultimately, because we used the ECHO model, we were able to obtain certification through the University of New Mexico—Project ECHO for a fee. While offering CE credits is highly attractive to our program participants, it raises important questions about gatekeeping in knowledge generation. A fundamental principle of CoP and ECHO is mutual learning and knowledge-sharing among participants. However, our experience underscores how certain sources of knowledge—particularly those tied to academic institutions—are often privileged over others. Despite being a community-based organization with over 20 years of experience providing direct HIV services to African immigrants, MAC was unable to obtain CE certification independently. Instead, we had to rely on external partnerships, highlighting the systemic barriers that favor traditional academic credentials over community expertise.

Another significant challenge was the limited number of African immigrant providers in HIV or related fields, such as mental health and women’s health. Early AI Health participants specifically requested guest speakers of African immigrant background; a need that was initially difficult to meet but was gradually addressed over time. Although the demographics of HIV care providers are slowly diversifying, the underrepresentation of first- and second-generation African immigrants continues to affect the cultural relevance and quality of care for this population. This systemic underrepresentation of African immigrant providers stems from structural barriers in medical education and training, such as the extensive U.S.-based residency and examination requirements imposed by American specialty boards. These processes disproportionately disadvantage first-generation African immigrant physicians, despite their qualifications to practice medicine, including HIV care. Legal reforms to address these barriers are underway, such as the HB 1129 in Washington which was signed into law in 2021 and the Physician Pathway Act in Massachusetts which was passed in 2024 [44,45].

Lastly, a challenge we experienced was meeting participants’ request for ongoing learning and connectedness. Suggestions were extending the program’s reach to include events, additional programs, and webinars that could further engage participants and broaden the impact of the community of practice. While many participants expressed a strong desire to continue learning from one another after completing the program—some even reapplying to join subsequent cohorts—the program’s current structure and funding did not support such continuity. This limitation highlighted the need for a shift from one-time, didactic trainings to more longitudinal models that better sustain engagement and learning. The ECHO model and the CoP framework are designed as longitudinal programming. Integrating application-based evaluations and peer-to-peer learning is essential for addressing the needs of both the workforce and the communities they serve. Although implementing longitudinal programming requires additional time and resources, successful AIDS Education and Training Center models exist that demonstrate the feasibility and impact of this approach [46,47]. Lessons from these models could inform adaptations to this program, emphasizing sustainable learning frameworks and ongoing professional development.

### Limitations

This evaluation has several limitations. First, we did not assess CtC implementation beyond the designated implementation period and therefore cannot determine whether changes were sustained over time. It is also possible that some participants met their CtCs after the program concluded, but delayed implementation was beyond the scope of our evaluation and was not captured. However, outcomes highlight the immediate benefits of CtC implementation and point to the potential value of sustaining such efforts. Relatedly, as we have argued, significant change needs to occur at organizational and structural levels, and most CtCs addressed individual or interpersonal levels of change. We did note that as more administrators and program managers joined in latter cohorts, the chosen CtCs, as well as case presentations, also shifted to the higher levels of change. Future programming should intentionally consider how to better support change at these higher levels. Second, while this analysis drew on programmatic data, many data points were self-reported and therefore subject to social desirability bias, even though they were collected anonymously. In addition, we did not measure downstream patient-level outcomes, although increasing provider capacity constitutes a significant outcome. Third, a significant portion of the data, particularly in earlier cohorts, had missing values, which may affect the generalizability of some findings. Fourth, further limitations are introduced by the use of ECHO, CoP, and CtC in this program, specifically in terms of evaluation and downstream outcomes. For example, the ECHO model relies largely on participation data and self-reported outcomes rather than demonstrating impact through patient-level health or behavioral changes [46]. A specific ECHO program is also hard to replicate given the need to adapt to the contexts of participants and their patients, which can vary widely across populations [48,49]. Finally, the differences in program structure over the years may complicate direct comparisons across cohorts.

## 5. Conclusions

AI Health was successfully developed through a community–clinical effort using an iterative and participatory process that engaged diverse community members, clinical providers, and social service professionals. Several evidence-based frameworks were used to create a program with a collaborative environment and measurable outcomes for changes in participants’ practices and organizational processes. Despite challenges and limitations, the structure and content of AI Health offers valuable insights to inform and guide other provider training programs aimed at improving services and health outcomes for racialized and marginalized populations. Future iterations of AI Health should incorporate longitudinal follow-up and include measures of service delivery and patient outcomes to better assess sustained impact. In addition, the program’s framing and analysis would benefit from engagement with structural determinants of health, migration, and decolonization scholarship to more fully capture the systemic and structural factors shaping HIV care for African immigrant communities. Strengthening institutional supports and creating pathways for organizational and policy-level commitments will also be critical to advancing systemic change alongside provider-level individual and interpersonal practice improvements. Ultimately, sustained investment in such innovative and inclusive approaches is essential to creating a more equitable healthcare system that effectively meets the needs of all communities.

## Figures and Tables

**Table 1:** AI Health Program Structure Over Time.

	Cohort 1	Cohort 2	Cohort 3	Cohort 4	Cohort 5
Timeline	8 January 2020 to 24 June 2020	2 April 2021 to 28 May 2021	24 March 2022 to 27 May 2022	20 April 2023 to 16 June 2023	4 April 2024 to 31 May 2024
Sessions	Twelve sessions, biweekly	Eight weekly sessions with an implementation week at the conclusion of the first 4 weeks	Two-day intensive and 8 weekly ECHO sessions		
Sessions Content	Didactic and ECHO case presentation	Didactic and ECHO case presentation	Didactic presentations followed by weekly ECHO sessions and CtC		
Evaluation Metrics	Pre CtC survey after every session, post CtC survey sent four weeks after each CtC was developed and focus group	Pre CtC survey after each session, post-CtC at the end of session 4 and session 8, and focus group	Pre CtC survey after each day of the intensive, post CtC survey at end of program, and focus group	Pre CtC survey after each day of the intensive, ECHO session evaluation, and post CtC survey at end of program	Pre CtC survey after each day of the intensive, ECHO session evaluation, post CtC survey at end of program and focus group

**Table 4:** CtC Socioecological Categories, Themes, and Illustrative Examples.

Categories	Theme	Theme Description	Illustrative Examples
Individual	Skill Building	Developing and enhancing practical abilities and techniques to improve service delivery and interactions with clients.	“How to better understand a client’s perspective on their mental health with asking questions that value their belief systems/culture”
	Knowledge Building	Activities and efforts aimed at increasing one’s understanding and awareness of specific topics or issues.	“Study more on cultures and not just racial challenges”
	Job Development	Identifying and pursuing career opportunities that align with personal interests and professional goals.	“After participating in today’s session, i would like to identify an organisations to work for that is line with HIV since that’s my area of interest”
	Knowledge building-Resources	Activities aimed at identifying, researching, and utilizing available resources to better support clients and enhance service delivery.	“I am going to explore more probono services and continue staying up to date with the laws and eligibilities that are available by creating a network of Providers and mobilize and advocate for the community”
	Relationship Building	Establishing and nurturing connections with clients to foster trust, collaboration, and effective communication.	“Conduct outreach to engage more African Members”
	Attitude Change	Shifting one’s perspective, beliefs, or mindset to better understand and relate to clients and their experiences.	“To be a voice for those who cannot advocate for themselves”
Interpersonal	HIV Care Practices	Within teams or among co-workers, activities and approaches aimed at promoting effective clinical and supportive practices to manage and treat HIV.	“Educate my clients who plan on getting pregnant vital reproduction info”
	Stigma	Efforts to reduce and dispel the negative attitudes and misconceptions associated with HIV	“Addressing HIV/AIDS stigma through awareness, that it’s not a specific type of person diseases”
	Patient/Provider Relationship	Interaction and rapport between patients and providers to enhance care quality and patient trust.	“Be sensitive and emphasize with clients experience”
	Internal Communications and Collaborations	The exchange of information, ideas, and research among healthcare team members to improve patient care.	“Ensuring staff are aware of how stigma influence care for African Immigrants”
Institutional	Resource Identification and Utilization	Activities focused on locating, identifying, and making use of various resources to support institutional goals and improve service delivery	“ I will gather information on services provided to AI within our program as well as review available data for Maryland”
	Systems/Process Change	Modifying or improving existing systems and processes within an organization to enhance relevance, efficiency and effectiveness.	“Developing appropriate language in considerations of one’s culture when discussing mental illness”
	Program Development	Designing, planning, and implementing programs to address specific needs or issues, ensuring they are effective and culturally sensitive.	“Post-Partum class that addresses reproductive health”
	Organizational Capacity Building	Efforts to strengthen an organization’s abilities, skills, and resources to improve performance and achieve its objectives.	“New way of outreach”
Community	HIV Epidemiological Profile of African Immigrants	Collecting, analyzing, and sharing epidemiological data specific to HIV prevalence and incidence among African immigrant populations to inform diverse individuals and organizations and guide interventions.	“Share the national data on AI HIV epi with key stakeholders”
	Communication and Connecting	Enhancing relationships and communication with various community leaders and organizations to support the African immigrant community.	“Collaborate to raise awareness of HIV and Hepatitis in the African Immigrant communities, especially the newcomers”
Public Policy	Policy Change	Identify key policy gaps and work collaboratively to create changes at the policy level that improve the lives of AI members affected by HIV.	“Draw up a policy proposal to increase undocumented AI engagement with health services”
	Advocacy	Activities that bring together community leaders, healthcare professionals, policymakers, and members of the African immigrant community to address the unique challenges faced by African immigrants living with HIV.	“To strive to advocate for the inclusion of linguistically appropriate interventions in the State and local health departments viral hepatitis program”

**Table 5:** CtCs Frequencies by Socioecological Level and Themes for the Period 2020–2024.

Categories ^b^	Theme	Cohort 1, 2020 n (%) ^a^	Cohort 2, 2021 n (%)	Cohort 3, 2022 n (%)	Cohort 4, 2023 n (%)	Cohort 5, 2024 n (%)	Total N (%)
ID	Skill Building	4 (3)	7 (15)	12 (10)	3 (6)	10 (17)	36 (9)
	Knowledge Building	25 (21)	8 (17)	7 (6)	9 (18)	1 (2)	50 (13)
	Job Development	0	2 (4)	0	0	0	2 (1)
	Knowledge building-Resources	10 (8)	0	9 (8)	3 (6)	10 (17)	32 (8)
	Relationship Building	1 (1)	0	6 (5)	0	2 (3)	9 (2)
	Attitude Change	8 (7)	4 (9)	17 (15)	5 (10)	9 (16)	43 (11)
IP	HIV Care Practices	5 (4)	0	1 (1)	1 (2)	1 (2)	8 (2)
	Stigma	1 (1)	0	1 (1)	0	0	2 (1)
	Patient/Provider Relationship	15 (13)	11 (23)	3 (3)	3 (6)	0	32 (8)
	Internal Communications and Collaborations	19 (16)	3 (6)	8 (7)	5 (10)	3 (5)	38 (10)
IT	Resource Identification and Utilization	7 (6)	0	0	1 (2)	0	8 (2)
	Systems/Process Change	5 (4)	5 (11)	6 (5)	3 (6)	2 (3)	21 (5)
	Program Development	4 (3)	4 (9)	11 (9)	1 (2)	4 (7)	24 (6)
	Organizational Capacity Building	5 (4)	0	5 (4)	2 (4)	0	12 (3)
CO	HIV EPI Profile of AI	2 (2)	0	0	0	0	2 (1)
	Communication and Connecting	8 (7)	2 (4)	12 (10)	9 (18)	5 (9)	36 (9)
PP	Policy Change	0	0	2 (2)	2 (4)	0	4 (1)
	Advocacy	1 (1)	0	2 (2)	1 (2)	0	4 (1)
NC	Not Clear	0	1 (2)	15 (13)	1 (2)	11 (19)	28 (7)

^a^ Percentage not always equal exactly to 100 due to rounding. ^b^ Full names of all abbreviations used: ID = Individual Socioecological Level. IP = Interpersonal Socioecological Level. IT = Institutional Socioecological Level. CO = Community Socioecological Level. PP = Public Policy Socioecological Level. NC = Not Clear. The response was either unclear or lacked sufficient detail for proper categorizing.

## Data Availability

The original contributions presented in this study are included in the article. Further inquiries can be directed to the corresponding author(s).
